# Interventional Management of a Rare Combination of Nutcracker and Wilkie Syndromes

**DOI:** 10.3390/jpm12091461

**Published:** 2022-09-06

**Authors:** Mihai-Claudiu Ober, Florin-Leontin Lazăr, Alexandru Achim, Dacian Călin Tirinescu, Gregor Leibundgut, Călin Homorodean, Maria Olinic, Horea Laurențiu Onea, Mihail Spînu, Dan Tătaru, Bogdan Săbiescu, Dan-Mircea Olinic

**Affiliations:** 1 Department of Interventional Cardiology, Medical 1 Clinic, University of Medicine and Pharmacy “Iuliu Hatieganu”, 400347 Cluj-Napoca, Romania; 2 Klinik für Kardiologie, Medizinische Universitätsklinik, Kantonsspital Baselland, 4410 Liestal, Switzerland; 3 Nephrology Department, “Iuliu Hatieganu” University of Medicine and Pharmacy, County Emergency Hospital Cluj-Napoca, 400347 Cluj-Napoca, Romania

**Keywords:** nutcracker, Wilkie, endovascular treatment, left renal vein compression, duodenal compression, mesoaortic angle, hematuria

## Abstract

Nutcracker and Wilkie syndromes are rare mesoaortic compression entities, and their association is even less common. Data on interventional treatment of these pathologies are still scarce, but results from limited case series are encouraging. We report the case of a previously healthy 45-year-old woman diagnosed with nutcracker and Wilkie syndromes who presented with macroscopic hematuria, intermittent pain in the left flank and hypogastric region, postprandial nausea, and unexplained significant weight loss. A successful endovascular approach with stent implantation in the left renal vein was performed, but the stent migrated toward the left kidney, and this acute complication was managed through an interventional strategy as well. At the three-month follow-up, the patient described a marked improvement in all symptoms, except for the macroscopic hematuria. As it was our strong belief that the approach was efficient, we further investigated the “hematuria”, which eventually led to the diagnosis of endometrial carcinoma. A hysterectomy and bilateral adnexectomy were planned, and chemoradiotherapy was initiated with the goal of preoperative tumor reduction. To our knowledge, this is the first reported case in which both Wilkie and nutcracker syndromes were effectively treated by stent implantation in the left renal vein, complicated with very early stent migration due to inadequate apposition to the less compliant venous lumen. The treatment of the duodenal compression was indirectly included in the stenting of the left renal vein, as reclaiming the venous lumen widened the aortomesenteric angle. The aim of this review is to discuss our center’s transcatheter experience with these rare disorders and explore the literature in order to establish the benefits and limitations of such an approach.

## 1. Introduction

A reduced angulation of less than 22 degrees between the abdominal aorta and the superior mesenteric artery (SMA), with an aortomesenteric distance of less than 8 mm, is a very rare vascular alteration [1] which leads to a reduction in the aortomesenteric space and consequent compression of its structures, mainly of the left renal vein (nutcracker syndrome) and/or the duodenum (Wilkie syndrome) [2]. While each of these syndromes represent a rare finding, their association is even less frequent. Because the congenital form is not as common, several acquired etiologies have been described, including rapid weight loss, compression by adjacent lymphadenopathy or malignancy, severe lordosis, pregnancy, or intestinal malrotation [3]. Nutcracker and Wilkie syndromes share a common etiology, namely weight loss.

The precise epidemiology of nutcracker syndrome is unknown, partly because of an absence of definitive diagnostic criteria and partly because of the variability in symptomatic presentation. However, unexplained hematuria is a common symptom and nutcracker has been diagnosed by doppler ultrasound in 40% of patients with this clinical presentation [4]. There may be left flank pain, usually associated with hematuria and sometimes accompanied by albuminuria and pelvic congestion (characterized by symptoms such as dysmenorrhea, dyspareunia, lower abdominal pain, dysuria, pelvic, vulvar, gluteal, or femoral varices, and emotional disturbance). Compression of the left renal vein (LRV) can cause reflux from the left renal to the gonadal vein, leading to lower limb varices and varicoceles in men. Although it is primarily a vascular disease, manifestations are predominantly urologic or gynecologic, yet some patients are also treated by vascular surgeons when lower limb varices are the chief complaint. Interestingly, a recent study retrospectively analyzed the data from high-definition renal computed tomography (CT) in 324 normal asymptomatic patients and identified an aortomesenteric angle < 41° in 30.5% of patients, with a greater prevalence in women, but an LRV ratio ≥ 4.9 in just 0.7% of the cases [5]. This may explain that some acute angles do not lead to clinical manifestation or pathological diagnosis of the two syndromes and remain only as an anatomical variant. This is sometimes called the nutcracker *phenomenon* and the term *syndrome* is reserved for patients with distinctive clinical symptoms associated with verifiable nutcracker morphologic features. The veins physiologically draining into the left renal vein include the left gonadal vein, left ureteral vein, left inferior phrenic vein, and left adrenal veins [6]. In nutcracker syndrome, these vessels are often engorged due to the decreased outflow of the left renal vein. These collaterals cause increased pressures in the gonadal vein, which causes increased pressures in the smaller and more fragile vesicular veins and pampiniform plexus, leading to varicocele development. Finally, because collaterals frequently fail to decompress the stenosed renal vein, hematuria results from blood transposition over fragile renal sinusoids into the collecting system [3]. Based on renal vein pressures, the patients’ strong collaterals are likely decompressed sufficiently enough to avoid hematuria, and vice versa. The treatment must therefore be tailored from case to case.

The symptomatology in both syndromes is nonspecific and is common to many other abdominal pathologies [2]. In nutcracker syndrome, the hallmark clinical symptoms (hematuria, proteinuria, and flank/pelvic pain) occur only in the presence of hemodynamically significant LRV stenosis leading to venous hypertension [7]. Macroscopic or microscopic hematuria appears as a result of the rupture of intrarenal varices triggered by venous congestion [8], which also induces an immune cascade in the vessel wall and consequently causes a greater release of norepinephrine and angiotensin II upon standing, leading to orthostatic proteinuria [9]. The numerous communications of the LRV with the lumbar venous plexus, inferior vena cava, and the left gonadal vein may explain the pelvic pain seen in advanced cases, as these venous systems become dilated as a result of renal venous congestion [10].

On the other hand, the compression of the duodenum results in non-specific gastrointestinal symptoms, such as nausea, early satiety, abdominal pain and vomiting, all of which are aggravated by eating [7]. These manifestations promote weight loss, which triggers further subsequent mesenteric fat loss, further reducing the aortomesenteric angle, thus resulting in a pathological circle [11]. Congenital factors include an abnormally high origin of the ligamentum Treitz, which draws the duodenum to the root of the mesentery. A short aortomesenteric distance or narrow aortomesenteric angle is a feature of the SMA, with duodenal obstruction leading to postprandial abdominal pain relieved by vomiting. The available therapeutical strategies consist of a high-caloric diet, various surgical approaches, and endovascular stenting, which not only restores the regular LRV flow, but also has the potential to correct the aortomesenteric angle, thus reducing the duodenum compression as well [12]. 

The anatomic disposition was first expressed as analogous to a “nutcracker” in 1937 by Grant [13] and the first venographic study of a patient with this clinical syndrome was reported by Schepper [14] in 1972. There are only a few reported cases in the literature that describe patients with radiological evidence of the compression of both the duodenum and the LRV, with even fewer patients reported to have both the radiological and clinical evidence for both disorders. Our PubMed/MEDLINE review of the literature found eight case reports describing concomitant nutcracker and Wilkie syndromes [15,16,17,18,19,20,21]. We hereby present our center’s experience with a 45-year-old female patient diagnosed with both Wilkie and nutcracker syndrome, presented with typical symptoms, treated with an endovascular approach, with a surprising outcome. The aim of this review is to discuss the advances in the diagnosis and treatment of these rare syndromes in order to better understand its outcomes and limitations.

## 2. Imaging Diagnosis

**Figure 1:**
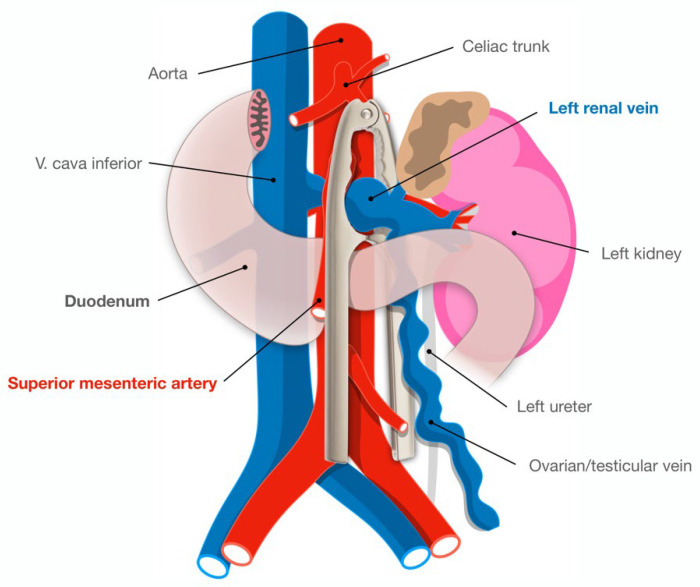
Illustration of the aortomesenteric angle in relation to the left renal vein and the terminal duodenum, tangling the two simulates the shape of a nutcracker (overlaid).

Normally the SMA emerges from the abdominal aorta at a 90-degree angle. The normal aortomesenteric angle is reported to be 28 to 65 degrees, and the normal aortomesenteric distance ranges from 10 to 34 mm [2]. The LRV is situated anterior to the aorta in the fork between the SMA and abdominal aorta. In anterior nutcracker syndrome, the SMA arises from the aorta at an acute angle, compressing the LRV and causing left renal venous hypertension and/or final part of duodenum (Figure 1). In posterior nutcracker syndrome, the LRV has a course posterior to the aorta and is compressed between it and the vertebral bones. In combined nutcracker syndrome, the anterior branch of the duplicated LRV is constricted between the aorta and the SMA, while the posterior is squeezed between the aorta and the vertebral bones.

The “gold standard” for diagnosis remains phlebography, intravascular pressure measurement, and intravascular ultrasound through which the venous pressure gradient between the LRV, the inferior vena cava, and the renal vein diameter can be accurately identified [22]. But all of these dedicated invasive investigations are being conducted on the basis of a high suspicion or a provisional diagnosis, which is always firstly based on a non-invasive imaging work-up. In a patient with an unremarkable workup for the common causes of hematuria and/or flank pain, a multimodality approach of a Doppler ultrasound followed by either CT or magnetic resonance (MR) venography will often suggest the diagnosis of an abnormal mesoaortic angle.

Ultrasonography can often reveal the compressed renal vein and allow for the measurement of the vein diameter and stenosis secondary to the compression. The long axis color Doppler view can exemplify the presence of a velocity gradient from the perihilar to mesoaortic region [22]. The presence of an increased flow through the collateral veins is further proof of renal venous hypertension. Although ultrasound can nicely display the stenosis and collaterals, studies have shown a poor correlation between the Doppler and the gold standard of the renocaval pullback invasive pressure measurements [23,24], which we consider to be the basis of the variation in the collateral decompression of the renal vein. For this reason, a direct measurement of the pressure between the left renal vein and inferior vena cava remains valuable for securing a diagnosis of nutcracker syndrome, with a pressure difference greater than 5 mmHg considered significant [22].

CT and MR angiography tests allow for the highlighting of the mesenteric artery origin from the abdominal aorta and the compression and stenosis of the LRV. Coronal and sagittal reconstructions also allow for the depiction of the left gonadal vein and collateral circulation with the lumbar veins. With sagittal reconstruction, it is possible to evaluate the incriminated angle, which if it is less than 35 degrees, is compatible with the diagnosis of the compression of the LRV and further clinical and physiological assessment should define if the physician is facing a *syndrome* or just a nutcracker *phenomenon*. Finally, an LRV diameter ratio (hilar to aortomesenteric ratio) of more than 4.9 has a positive predictive value of 100% [24].

## 3. Center Experience

### 3.1. Case Report

**Figure 2:**
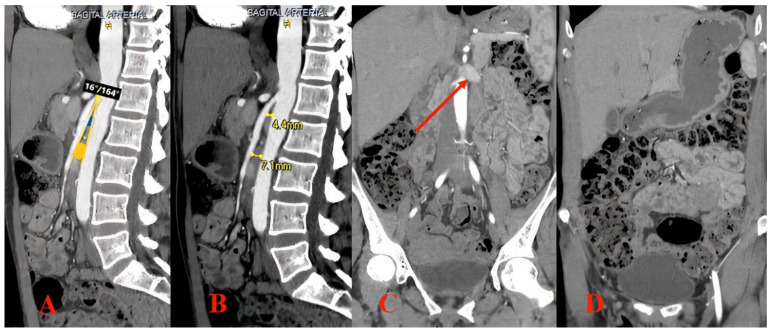
Computed tomography (CT) angiography findings. (**A**) Sagittal view revealing an aortomesenteric angle of 16 degrees; the blue circle represents the left renal vein and the yellow circle represents the location of the terminal duodenum. (**B**) Sagittal view revealing an aortomesenteric distance of 4.4 mm at the renal vein level and 7.1 mm at the duodenum level. (**C**) Coronal view showing a dilated left renal vein (arrow). (**D**) Coronal view showing an important distention of the stomach, with gastric stasis and gas distension of intestines.

A 45-year-old female with no medical history who was recently diagnosed with nutcracker syndrome in the nephrology department was referred for further investigations and treatment strategy decision making. She complained of macroscopic hematuria, intermittent pain in the left flank and hypogastric region, and postprandial nausea and described a significant weight loss of 10 kg in the last 3 months. After the initial differential diagnosis of macroscopic hematuria, urologic and general nephrological causes were excluded (renal biopsy revealed no pathologic changes on light microscopy and immunofluorescence). The subsequent computed tomography (CT) angiography (Figure 2) confirmed the presence of nutcracker syndrome with an aortomesenteric distance of 7.1 mm and reduced angulation of 16 degrees, with an important dilation of the left renal vein. In addition, severe distention of the stomach and intestines was noted.

Notably, a tumor growth was described in the right ovary, possibly a hypertrophic corpus luteum, with a gynecological recommendation for periodic follow-up.

**Figure 3:**
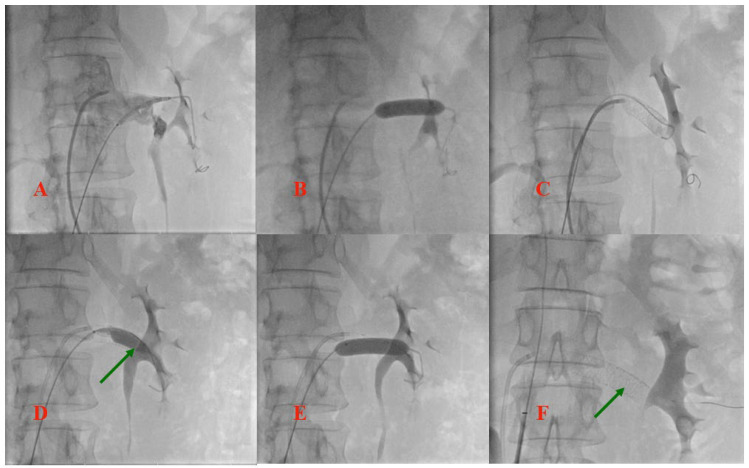
Left renal vein angioplasty-initial procedure. (**A**) Stent placement at the ostium of the LRV. (**B**) Stent inflation. (**C**) Stent partially migrated toward left kidney. (**D**) Stent repositioning using a partially inflated balloon. (**E**) Stent repositioned at the ostium of the vessel and balloon inflation (arrow). (**F**) Final result, with the stent partially migrated again towards LRV (arrow).

**Figure 4:**
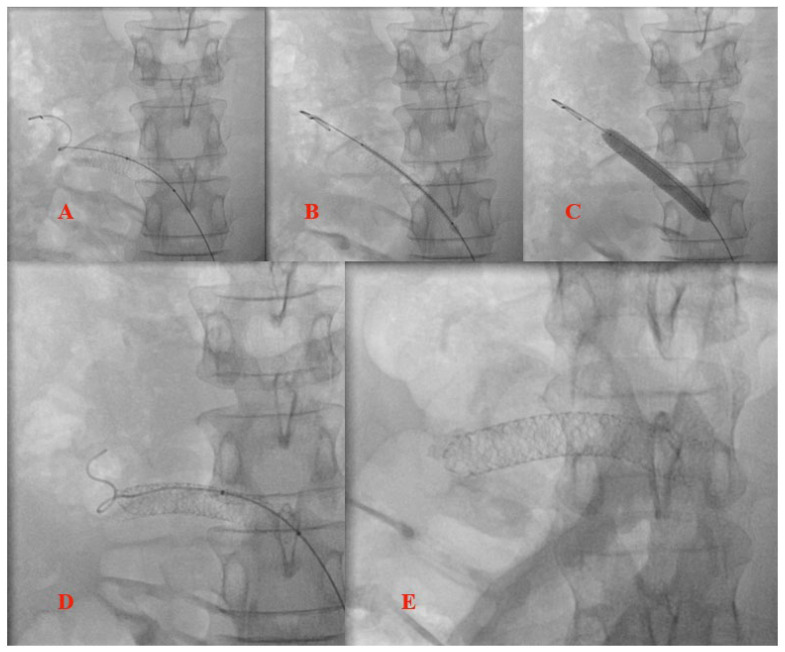
Left renal vein angioplasty optimization of the initial angioplasty. Note that the images are mirrored because the patient was positioned upside down on the operating table for jugular access. (**A**) Initial stent repositioning and post-dilation. (**B**) Second stent positioning and (**C**) stent deployment. (**D**) Second stent post dilation. (**E**) Final result.

Selective left renal vein angiography was further performed and revealed the obstruction at the junction of the LRV with the inferior cava. Since the patient refused all surgical options, the interventional strategy consisting of LRV stenting was planned for the same procedure, with the aim of not only restoring normal LRV flow but also correcting the aortomesenteric angle, thereby reducing the compression of the duodenum. Bilateral femoral vein access was obtained and two wires were crossed in the LRV (a diagnostic super stiff Amplatz wire from the right and a coronary BMW wire from the left, as landmark). A balloon expandable stent measuring 9/40 mm was implanted at the obstruction site, but the stent migrated towards the renal side of the vein when post-dilation was tented. At this point, the guide catheter was replaced with a long-armed sheath and the stent lumen was recrossed with a super stiff wire, managing to retrieve the stent at the ostium vessel with a partially inflated balloon. Post-dilation was attempted once more, but, due to the trend towards migration, the procedure was aborted. As a post-procedural vascular ultrasound showed partial migration of the stent toward the left kidney and the patient complained of persistent flank pain, a second procedure was planned the next day, using the jugular vein as the access site, with the aim of repositioning the stent and thus optimizing the result. Echo-guided access to the jugular vein was obtained and the stent lumen was crossed with a super stiff wire. A 10/30 mm balloon was advanced into the stent, with 5 mm protrusion distally to the stent. After partial inflation of the balloon, the retraction of the stent at the lesion site was possible and a complete inflation at 16 atmospheres was performed, with a good apposition to the vessel wall being obtained, but with important deformation of the distal struts of the stent. As a result, a second balloon expandable to 9/60 mm was implanted at the ostium, which partially overlapped with the previous stent, and post-dilations with a 10/30 mm balloon were performed along the entire length of the stents. Final post-dilatation at the ostium was performed with a 12/40 mm balloon, with a good final angiographic result and a good and stable apposition being obtained, as the compression of the vein was no longer visualized. Figure 3 and Figure 4 illustrate the main steps of the endovascular procedures.

After the procedure, no complications occurred and the patient reported the relief of their pain, but with persistent hematuria. Dual antiplatelet therapy was initiated (Aspirin and Clopidogrel) for 3 months, as well as Sulodexide therapy. The patient was discharged the next day and clinical and imagistic follow-up was scheduled. As per the Wilkie syndrome, the patient was encouraged to regain weight by hypercaloric intake and enriched fluids (if tolerated), and adopt an antalgic position that has been described to aid enteral feeding (such as the prone position, left lateral decubitus position, and the knee–chest maneuver). Moreover, the treatment of the duodenal compression was indirectly included in LRV stenting treatment as reclaiming the venous lumen widens the aortomesenteric angle (the renal vein being superior in relation to the duodenum, closer to the corner of the angle).

**Figure 5:**
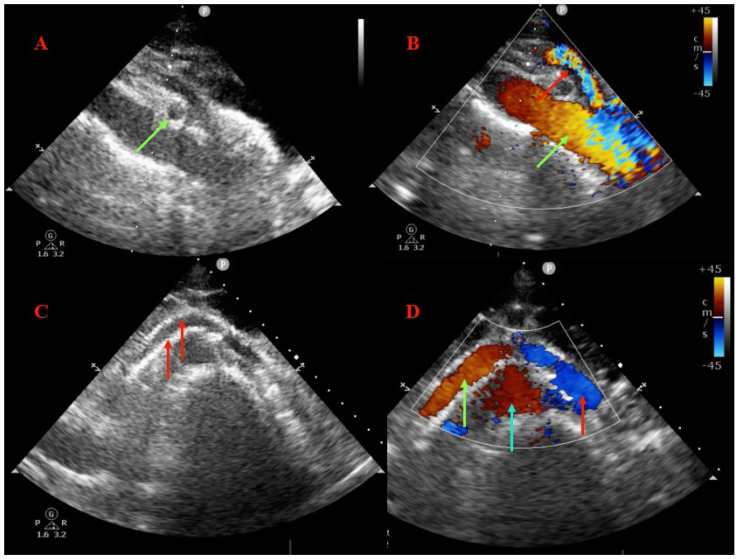
Abdominal ultrasound findings at the three-month follow-up. (**A**) LRV short axis showing well-expanded and well-apposed stent (green arrow), with enlarged aortomesenteric space. (**B**) Doppler ultrasound showing regular aortic (green arrow) and mesenteric (red arrow) flow, with enlarged aortomesenteric space. (**C**) LRV long axis confirming good expansion and apposition of the vein stent (red arrows). (**D**) Doppler ultrasound confirming regular flow in the LRV (green arrow), inferior vena cava (red arrow), and aortic artery (blue arrow).

A follow-up was conducted after 3 months. At this point, the patient described an important improvement of symptoms, with complete relief of their pain in the left flank, no postprandial nausea, and a slight weight gain (1.5 kg), but with persistent macroscopic hematuria. However, the patient also described frequent episodes of metrorrhagia, which raised an important question: is the hematuria described by the patient real or is it more? Thus, further investigations into the ovarian tumor were recommended and, after magnetic resonance imaging and another gynecological examination were performed, the diagnosis of endometrial cancer was established and chemoradiotherapy was initiated with the aim of pre-operative tumor reduction. An abdominal ultrasound showed the permeable stent in the correct position in the LRV, without restenosis or compression, and regular flow in the LRV (Figure 5). Another clinical and imaging follow-up was scheduled after the planned surgery (a hysterectomy and bilateral adnexectomy). At the last telephone follow-up, the patient was noted to be continuing her chemoradiotherapy, with the indication for surgical resection depending on the progressive oncologic status, and that the hematuria had ceased and the urine was subjectively clear.

### 3.2. Stent Migration

We described the case of a 45-year-old woman with symptomatic nutcracker and Wilkie syndromes diagnosed by CT angiography, without any other known comorbidities. Because the patient refused surgery, an interventional approach was planned, which consisted of a stent implantation in the LRV. Although nutcracker syndrome is a well-characterized pathological condition, there is no clear consensus on the optimal treatment approach. Endovascular treatment represents a valid alternative for surgery, as reported by several authors [25,26]. However, this strategy is associated with a non-negligible risk of stent migration, as shown by several case reports and studies [27,28]. Wu Z et al. found a stent migration rate of 6.7% in a cohort of 75 patients, with no significant difference in the preoperative anteroposterior diameter and peak velocity of the aortomesenteric portion, and renal hilum of the LRV on a duplex ultrasound [28]. The authors concluded that the stent selection and its accurate deployment are important factors in preventing stent migration. Another general recommendation to avoid this complication refers to oversizing the stent by 20% and placing it in the first branch of the renal vein [29]. These aspects must be carefully calculated in advance, as there have been cases of stent migration into the right atrium that required open-heart surgery [26].

In our case, we chose the largest available stent in our clinic, which was a 9/40 mm Zeus CC balloon expandable stent (Rontis AG, Zug, Switzerland) and inflated it at high pressure, which provided a 10–15% oversizing, but a stent migration toward the left kidney still occurred. The complication was managed by retrieving the stent using a partially inflated balloon and implanting a second longer stent which was partially overlapped with the previous stent, significantly increasing the stability. The short-term follow-up demonstrated the preserved patency and correct position of the stent; thus, it is fair to assume that not only a diameter oversizing but also a length oversizing are important factors to be considered in order to prevent stent migration.

Moreover, as all of the symptoms except for the “hematuria” were significantly improved, it is our strong belief that the intervention was efficient, and the bleeding might actually be metrorrhagia, determined by the endometrial cancer. Furthermore, the oncological disease was most likely the main trigger of the weight loss, which could have determined the reduction in the aortomesenteric space and consequently in the compression of its structures.

Regarding the post-procedural treatment, the data are still scarce and, taking into consideration the active bleeding as well as the high ischemic risk (a complex procedure followed by a complication, and the long stent length), we arbitrarily decided to treat this patient with 3 months of dual antiplatelet therapy. The role of antiplatelet and anticoagulant agents in the prevention of venous in-stent restenosis and thrombosis requires further investigation, with particular attention on a unique subset of patients (after thrombosis vs. nonthrombotic vein lesions) [30,31].

Finally, the disappearance of the metrorrhagia during chemoradiotherapy supports our theory of treatment efficacy in terms of complete symptom relief and renal vein decompression. The uterine tumor complicated this case by “double jeopardy”, perhaps causing the tapering of the meso-aortic angle and further mimicking the hematuria.

## 4. Surgical or Interventional Management

Patients with mild symptoms can be treated conservatively, with emphasis on weight gain that increases retroperitoneal adipose tissue, resulting in a change in the position of the left kidney with a decrease in tension on the LRV; this approach has been shown to relieve symptoms of nutcracker in 30% of patients [22]. This is highly encouraged in young individuals (those <18 years) as body growth releases the LRV from the arterial fork.

As per open surgical treatment, various techniques have been employed in an attempt to either decrease venous hypertension or alleviate the predominant symptoms of hematuria or pelvic congestion. The severity of symptoms, patient demographic, and the level of understanding of the available local expertise techniques can guide the clinician as to when and how to intervene. The first reported case of treated nutcracker was in 1974 [32]. Since then, a number of surgical approaches have been described. These include direct reimplantation of the left renal vein [33], SMA transposition [34], nephropexy [35], autotransplantation of the left kidney [36], isolated nephrectomy [37], and external stenting or “shielding” of the LRV [38]. Most of these approaches were developed to lower venous hypertension. Some techniques, such as left gonadal vein ligation, gonadocaval bypass, splenorenal venous bypass, and/or embolization and sclerosis of pelvic varices, are more directed to treat pelvic congestion symptoms [39]. The ligation of the collateral vessels, coil embolization of the gonadal veins, and ablation of the varices have been established together with renocaval pressure gradient relieving procedures in cases where pelvic congestion resulted from nutcracker syndrome [40].

Endovascular stenting of the left renal vein has recently been documented, with multiple studies demonstrating symptoms relief [25,26,27]. In fact, endovascular treatment could also solve the problem of duodenal compression (“2 in 1”), a great advantage over surgical reimplantation. The first stent was implanted in 1994 by Neste et al. [40] and since then, transcatheter treatment has gained popularity and notoriety due to its high success rate and few complications. The most relevant complication (for incidence and potentially damage) is stent migration, while in-stent restenosis and venous occlusion resulting from fibromuscular hyperplasia or thrombosis rarely occur. Thrombosis rather occurs before stenting, due to stasis in the dilated vein [41]. This detail is not negligible as the thrombus can embolize and cause acute pulmonary embolism [42]. There is no consensus on the post-interventional antithrombotic regimen but a “defensive” management would be 2–3 months of initial anticoagulation (until the stent endothelialization occurs) and prescribing aspirin long-term or dual antiplatelet therapy for at least 2 months [22,24]. The only limitation with endovascular stenting would be the lack of long-term data, although there is a signal of good long-term follow-up after 2–3 years [43]. The most pertinent reported cohorts of stented nutcracker syndrome patients are summarized in Table 1; at first sight, variability can be observed in the study population and follow-up. In one of the largest available registries to present, Chen et al. [26] retrospectively evaluated 61 patients with nutcracker syndrome treated by an endovascular approach and at long-term follow-up (66 months), there were only 2 patients without a significant improvement in the symptoms of hematuria, proteinuria, and flank pain, while the rest of the patients experienced clinical improvement at various periods of time (most of them after 6 months). Based on these observations, the authors recommended this approach as the primary option for nutcracker syndrome. The stenting techniques were mostly “borrowed” from the percutaneous experience in superior vena cava syndromes or May–Thurner syndrome (chronic compression of the left iliac vein against lumbar vertebrae by the overlying right common iliac artery) [44]. Due to the risk of migration, an oversized auto-expandable stent should always be preferred; they have more radial force and prevent recoil compared to dedicated venous stents. There is only a single published study that has implanted a specifically designed venous stent (Zilver Vena; Cook Medical, Bloomington, Ind) with relatively good outcomes, but in a small cohort (20 patients) with a short follow-up (10–122 days) [45]. Notably, a recent study from the University of Pittsburgh, USA used IVUS in 61% of their cases and had no stent migration, which highlights the importance of accurate stent sizing [46]. Interestingly, a group from China managed to build customized stents for patients with nutcracker syndrome by 3D printing; for the first step, they printed the entire kidney model based on CT images exported in DICOM format, then, through surgical planning, they finally printed out the stents using titanic alloy powder [47]. The only downside to this innovative idea is that the stent needs to be implanted surgically, as it is already expanded [47]. With time and more intensive imaging screening, it is likely that the prevalence of these syndromes will increase, which will inevitably refine our experience with these percutaneous techniques. The principle is the same as in coronary interventions: multidisciplinary teams, intraprocedural imaging, and proof of ischemia are encouraged [48]. In a patient who has a thin or asthenic body habitus, nutritional support can defer or replace surgical treatment. The final decision depends on the severity of symptoms. Certainly, the cause of weight loss must also be sought, because the nutritional vicious circle could have another origin.

## 5. Conclusions

Nutcracker and Wilkie syndromes are very rare findings, and their association is even less common. Interventional treatment of these pathologies is described in limited case series, and even if data are scarce, the results are encouraging and the latest techniques solve the problem of stent migration. To our knowledge, this is the first reported case in which both Wilkie and nutcracker syndromes were effectively treated by stent implantation in the left renal vein, complicated with acute stent migration that could also efficiently be treated by an endovascular approach. In contrast to surgical treatment, renal vein stenting can also relieve duodenal compression by opening the meso-aortic angle (because of the vein being located higher up, within the angle). On the basis of our case and several other case series reports, we believe that a tailored procedure consisting of a careful selection of a self-expandable stent oversized in length and diameter and close monitoring is a safe and effective strategy.

## Figures and Tables

**Table 1:** Studies of left renal vein stenting for patients with nutcracker syndrome; age and follow-up are mean values.

Study	Year	Cohort (Patients)	Age (Years)	Stent Type	Outcomes	Reintervention/Complications	Follow-Up (Months)
Chen et al. [49]	2005	3	10	Optimed (self-expandable)	100% stent patency at follow-up, resolution of hematuria	None	36
Hartung et al. [50]	2005	5	34	WALLSTENT	Symptoms resolution in all	2 patients presented stent migration after 3–4 months and recurrence of symptoms due to re-compression of the vein	14
Basile et al. [51]	2007	3	19	Luminexx (self-expandable)	100% stent patency at follow-up, resolution of hematuria	None	14–18
Chen et al. [26]	2011	61	26	WALLSTENT, SMART, Palmaz	Improvement of symptoms in 59/61 patients; 100% stent patency after 6 years, including the re-stented patients	2 stent migrations and reinterventions	66
Baldi et al. [52]	2011	2	50	SMART control	100% resolution of symptoms	None	12–24
Wang et al. [53]	2012	30	18	SMART control	100% stent patency at follow-up, resolution of hematuria	2 stent migrations (uneventful at follow-up)	36
Li et al. [54]	2013	3	16	Protégé	100% stent patency at follow-up, resolution of hematuria	None	6–60
Wu et al. [28]	2016	75	27	WALLSTENT, SMART control	3/5 patients who had stent migration developed symptoms again	5 stent migrations, from which 3 required open surgery	6–126
Policha et al. [55]	2016	3	33	WALLSTENT	100% stent patency at follow-up, resolution of hematuria	2 uneventful stent migrations	20
Avgerinos et al. [56]	2019	18	38	WALLSTENT, Protégé, SMART control, ev3, Zilver	72% symptoms resolution, 85% primary and 100% primary-assisted patency at 2 years follow-up	1 re-stenting, 2 balloon post-dilatations, 2 renal auto transplantations	41
Cronan et al. [57]	2021	10	16	Zilver, Venovo	70% symptoms resolution,	2 re-stenting with WALLSTENT for restenosis	3–37

## Data Availability

Not applicable.

## Abbreviations

SMA, superior mesenteric artery; CT, computed tomography; LRV, left renal vein; MR, magnetic resonance.
